# Measuring Changes in Upper Body Movement Due to Fasting Using a Camera

**DOI:** 10.3390/s24227242

**Published:** 2024-11-13

**Authors:** Longfei Chen, Muhammad Ahmed Raza, Imran Saied, Tughrul Arslan, Robert B. Fisher

**Affiliations:** 1 School of Informatics, The University of Edinburgh, 10 Crichton St, Edinburgh EH8 9AB, UK; longfei.chen@ed.ac.uk (L.C.); m.a.raza@ed.ac.uk (M.A.R.); 2 School of Engineering, The University of Edinburgh King’s Buildings Campus, The University of Edinburgh, Edinburgh EH9 3JL, UK; isaied@exseed.ed.ac.uk (I.S.); tughrul.arslan@ed.ac.uk (T.A.)

**Keywords:** fasting effects, upper body movements, video-based measurement, behavioral analysis

## Abstract

Understanding activity levels during fasting is important for promoting healthy fasting practices. While most existing studies focus on step counts to objectively assess the impact of fasting on activity levels and behavioral changes, the results have been mixed. Despite evidence showing that individuals spend a significant amount of time sitting while fasting, there has been no objective measurement of body movement or activity levels during sitting and fasting. This research employs a video-based, unobtrusive human body movement measurement system to monitor upper body movements during fasting and non-fasting periods over several days. Key movement features, such as inactivity, movement speed, and movement scale, were automatically extracted from the video monitoring data using a computer vision pipeline. These features were then statistically compared using *t* tests between fasting and non-fasting periods, analyzed by hour of the day and across different days. The results of the monitoring of five participants during typical daily sitting office work and fasting for 3–5 days indicate no consistent pattern of upper body movement changes due to fasting among the participants.

## 1. Introduction

Fasting, particularly during periods like Ramadan, has widespread implications not only for individual health but also for workplace productivity and well-being. It is mandatory for Muslim adolescents who have reached puberty and healthy adults to fast every day from sunrise to sunset during the ninth month of the Islamic calendar. They have to refrain from eating, drinking, and engaging in any other physical indulgences during this fasting period. While eating and drinking are permitted during the night, during Ramadan there are usually only two meals per day—one before sunrise and one after sunset—instead of the customary three or more meals, and there is no opportunity for mid-day snacks. This change in eating habits might, therefore, have a detrimental effect on energy intake, body weight, and hydration levels over the month [1,2]. Fasting, particularly for the whole day, also influences physical activity levels through various physiological and psychological mechanisms, including reduced energy, hormonal changes, altered metabolic rate, negative mood and mental states, and decreased motivation [3]. Understanding how fasting influences physical activity, particularly in sedentary office environments, can help shape both personal health practices and organizational policies. Monitoring fasting’s effect on movement and energy expenditure can provide objective data to inform workplace accommodations, such as adjusting work hours or physical activity recommendations during fasting periods.

How does fasting affect an individual’s physical activity? Previous studies have yielded mixed results. For example, subjective questionnaires and self-reported diaries [4,5,6] suggest that low to moderate daily activity remains relatively unaffected by Ramadan fasting. However, a decrease in high-intensity activities has been observed [4,7]. For instance, soccer players experience a significant reduction in running quantity during Ramadan, along with decreased aerobic capacity, speed endurance, and jumping performance [7]. But these findings were based on self-reported data, which may not accurately reflect true physical activity levels. Objective measurements using wearables in studies [8,9,10] primarily rely on step counts to assess physical activity levels, showing a reduction in step counts during Ramadan. While this method provides accurate data, it predominantly focuses on lower body movements, only partially capturing overall physical activity, and often overlooks the significant time individuals spend seated in office environments. During Ramadan fasting, most people spend substantial time sitting or lying down, sometimes exceeding 10 h per day [4,5]. There is currently a lack of objective, real-time monitoring systems that capture upper body movement during fasting, which is crucial for understanding its impact on productivity and health. The effect of fasting on physical activity during prolonged periods of sitting remains unclear. It would be valuable to investigate whether fasting influences activity levels while sitting, especially in typical daily environments, with a particular focus on upper body movements.

This research aims to address this gap by employing a non-invasive, vision-based system to evaluate how fasting affects upper body movement during typical office work, offering a more detailed understanding of fasting’s impact on physical activity in a sedentary setting. Compared to traditional wearables, the vision-based method is hands-free, easy to set up, and less invasive, allowing for natural observation without disrupting participants’ routines. It provides objective measurements at clinically acceptable accuracy [11], free from the bias associated with self-reporting [4], and captures comprehensive upper body movements without requiring multiple devices. This approach supports longitudinal tracking of changes across fasting and non-fasting periods.

**Figure 1:**
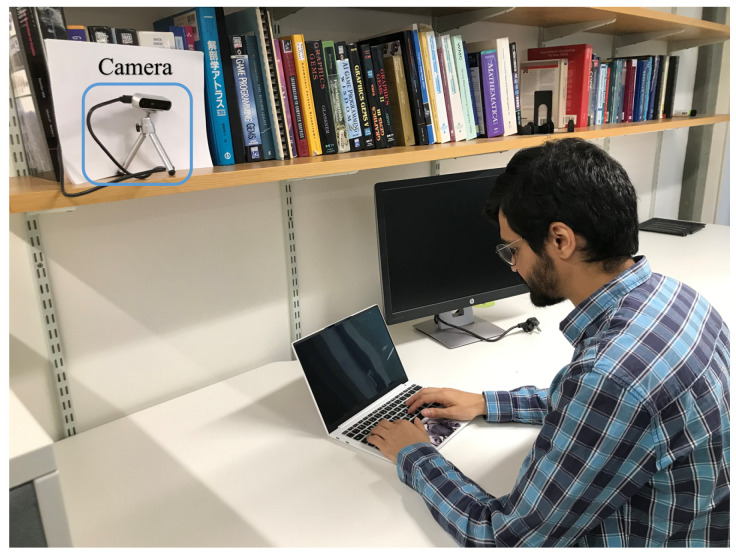
The RGB-D camera (Intel RealSense D415 [12]) monitors a participant during work in a university office. The camera captures upper body movements in real time, comparing these movements between fasting and non-fasting days. The visual data are processed immediately by a compact computer processor (Jetson Orin Nano [13]) to calculate metrics such as body movement speed, body movement scale, and body inactivity, providing insights into behavioral changes during fasting.

To achieve this, a vision-based human body motion measurement system consisting of a compact computer processor and an RGB-D camera was used to estimate upper body movements during fasting and non-fasting periods. The system monitored five participants’ body movements for 3 to 5 days during both fasting and non-fasting periods, and the differences were compared. Figure 1 illustrates the monitoring system alongside a participant. The experimental results show that fasting affects the movement patterns of the five participants differently. Two participants show statistically significant (p<0.05) yet opposite changes in movement, while the others do not, demonstrating substantial interpersonal variability.

## 2. Related Works

### 2.1. Effect of Physical Activity in Sports

Rebaï et al. [14] investigated the impact of Ramadan fasting on 18 healthy male soccer players. The tests were conducted in the afternoon on the 1st and 4th days of Ramadan, using the Wingate test to measure peak power (PP), mean power (MP), and the fatigue index (FI). Similarly, Aloui et al. [15] researched 12 healthy amateur soccer players to assess the effects of Ramadan fasting on their physical performance. Tests were performed in the morning on the 1st, 2nd, and 4th days of Ramadan, as well as after Ramadan. The physical tests measured repeated sprint ability (RSA), peak power (PP), total work (TW), and maximal voluntary contraction (MVC). Both studies revealed a significant decline in performance while fasting for soccer players.

Aziz et al. [16] examined the effects of Ramadan fasting on nine healthy, male Muslim athletes, with tests conducted in the morning and afternoon during the 1st to 4th days of Ramadan. The Wingate test and technical skill assessments were used to evaluate the athletes. The research found notable performance declines, with peak power (PP) and total work (TW) decreasing both in the morning and in the afternoon. Bouhlel et al. [17] investigated the effects of fasting on 10 male physical education students, with tests conducted in the afternoon on the 1st and 4th days of Ramadan. The physical tests measured sprint performance (for both arms and legs), vertical jump height (VJH), and handgrip force (HF). The research found significant performance declines, with arm-sprint and leg-sprint performance dropping significantly on the 4th day.

The findings indicate that the athletes experienced progressive declines in power and performance as Ramadan progressed. Although understanding the effect of Ramadan on the physical performance of sportspeople may be of importance for elite athletes, it does not show the impact of fasting on the daily lives and performance of normal individuals.

### 2.2. Subjective Measurement of Physical Activity

Al-Hourani et al. [5] observed 57 female subjects (mean age, 21.6 years) during Ramadan fasting. Each subject kept a 3-day activity diary, recording the type and duration of physical activity in the week before Ramadan and the second week of Ramadan. The results showed that the time spent lying down, sitting, and watching TV slightly increased to 7.82 ± 2.47 h per day, while the time spent on household chores decreased to 2.67 ± 1.47 h per day. However, none of these changes were statistically significant.

A similar activity pattern was recorded in the research conducted by Poh et al. [6], which found that among school children aged 10 to 13 years, boys spent more time in prayer compared to girls during Ramadan, but the time spent on all other activities did not differ between the fasting and non-fasting months.

Alwalweedi et al. [4] investigated the physical activity of 29 healthy participants in terms of frequency, duration, and intensity using the International Physical Activity Questionnaire (IPAQ) one week before, during, and after Ramadan fasting. There was a significant reduction in vigorous and moderate activity; however, walking activity did not show significant differences. Again, the average sitting time increased significantly to 13.21 ± 2.47 h per day during Ramadan.

However, the self-reported activity type and duration may not objectively represent the actual physical activity level. For example, questionnaires are limited by subjective measurement and desirability bias [4].

### 2.3. Objective Measurement of Physical Activity

Racinais et al. [8] investigated the activity patterns of 11 moderately active Muslim males by counting their steps using a hip-worn accelerometer for 3 days before, during, and after Ramadan fasting. A significant increase in step count around midnight (2–5 a.m.) was observed during Ramadan, which is not unusual, as that time is spent preparing Suhoor (eating before starting the next day’s fasting) time.

Geok et al. [9] also compared the step counts of 53 males and 54 females two weeks before, during, and after Ramadan, finding an average reduction of 1000 steps per day during Ramadan. A similar result was found in [18], which measured the monthly step counts of 16 females and 13 males, showing a significantly lower step counts during Ramadan.

Farooq et al. [10] conducted a large-scale study of 209 participants, comparing their step counts during Ramadan and the months before and after over a period of 7 years, covering 3 months each year. There was a reduction in daily steps during Ramadan (−385 ± 158).

Although step count is a widely used objective measure of activity level, it largely reflects lower body movement and may not account for activity levels involving the upper body. None of the existing studies has explored upper body movement during fasting, focusing on general daily activities. This research aims to use a vision-based motion measurement system to monitor upper body movements during daily sitting scenarios.

### 2.4. Energy Expenditure Measurement

During Ramadan, employees fast from dawn until sunset, with no food or drink intake during the day. Measuring energy expenditure in the workplace during this time provides critical insights that can help organizations optimize the work environment, enhance employee well-being, and maintain productivity.

Physical activity-related energy expenditure (PAEE) is the most variable component of total daily energy expenditure [19]. Indirect calorimetry (IC) and doubly labeled water (DLW) are two widely recognized and reliable methods, along with heart rate monitoring [20]. However, these methods often require specialized equipment and laboratory setups [21]. Another approach is to consider the type and duration of activities. The Metabolic Equivalent of Task (MET) is a useful tool for this purpose [22]. One MET represents the energy expenditure at rest, i.e., approximately 3.5 milliliters of oxygen per kilogram of body weight per minute (ml/kg/min); for example, light activities like sitting or slow walking have MET values between 1.1 and 2.9. Accelerometers are also commonly used to measure a person’s movement [22,23,24]; however, most of these devices primarily calculate step counts as well. In this research, a vision-based method is designed to objectively and non-invasively estimate energy expenditure indirectly by quantifying activity levels and duration.

## 3. Method

This research involved five participants (P1: M, 29; P2: M, 70; P3: M, 40; P4: M, 37; P5: M, 35; profiles shown in Table 1), who performed daily tasks while seated in various university offices. Specifically, three of the participants engaged in full daytime fasting without skipping a day during Ramadan, whereas two participants practiced intermittent full daytime fasting after Ramadan. Each participant was monitored for 3 to 5 days under both conditions. The monitoring process was completely unscripted and involved natural observation without disrupting participants’ routines. The monitoring dates were not necessarily continuous due to participants’ absences from the workplace (e.g., weekends, remote working days, etc.).

The vision-based system employs standard, off-the-shelf equipment, including an RGB-D camera (RealSense D415 [12]) and a compact computer processor (Jetson Orin Nano [13]). The camera is positioned on a shelf, approximately 1 m above the participants, to ensure full upper-body visibility (see Figure 1). It provides both color and depth images at 640 × 480 resolution and 30 frames per second and captures human movements in real time. Standard image processing functions, such as body region extraction and optical flow estimation for body movement measurement [25,26], are performed by the compact processor.

**Figure 2:**
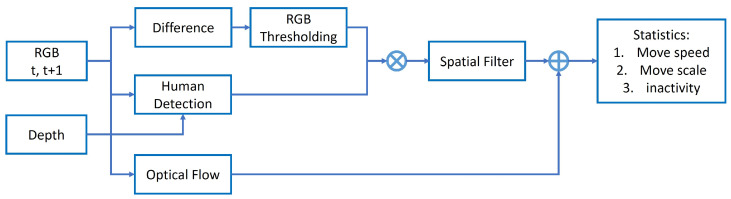
Block diagram for the monitoring system. Detailed calculations and parameters of these methods are described in [28].

Specifically, computer vision algorithms were employed, including non-parametric background modeling in the depth map [27] and human body identification in the foreground. This was followed by the detection of significant pixel variations on the human body based on changes in RGB color-channel values. Detailed calculations and parameters for these methods are described in [28]. Subsequently, the pixel-wise movement vector was computed using dense optical flow [29] applied to the significantly varying pixels extracted in the previous step. The method processes visual data at a rate of 7–8 frames per second, with no original images saved to protect privacy. For each frame, the 2D coordinates of the extracted human-body bounding box, the number of significantly varying pixels, and their respective 2D coordinates and optical flow were used to calculate movement statistics. The pipeline for the monitoring system that extracts body movement features is illustrated in Figure 2.

It achieves a 0% false positive rate for inactivity detection (with a ±3-frame temporal tolerance) [28]. Additionally, it attains a Root Mean Square Error (RMSE) of 0–2.5 pixels per frame for 2D human body movement speed estimation [30]. The camera’s sensors enable accurate identification of body regions and movements without requiring wearable devices, making the system non-interactive and unobtrusive.

To compare movement patterns between fasting and non-fasting periods and to explore potential changes in overall activity levels, three key performance metrics describing upper body movement were calculated:

**Inactivity:** The proportion of time during which no movement is detected while the person is within view is calculated as the number of frames with no detected body motion ($N_{\mathrm{inactive}}$) divided by the total number of frames with detected bodies ($N_{\mathrm{total}}$) captured during that time period. This metric measures the ratio of inactivity.

(1) $$\mathrm{Inactivity}=\frac{N_{\mathrm{inactive}}}{N_{\mathrm{total}}}.$$

**Movement Scale:** The ratio of the area occupied by moving parts of the body (defined by the bounding box of pixels with significant variations) to the total area of the body ($A_{\mathrm{body}}$—defined by the bounding box of the entire body). This metric indicates the proportion of the body that is actively moving.

(2) $$\mathrm{Movement}\;\mathrm{Scale}=\frac{A_{\mathrm{moving}}}{A_{\mathrm{body}}},$$

where $A_{\mathrm{moving}}$ is the area of the bounding box surrounding the pixels with significant variations across all RGB channels on the human body [28] and $A_{\mathrm{body}}$ is the area of the bounding box surrounding the entire body detected by the YOLO object detector [31].

**Figure 3:**
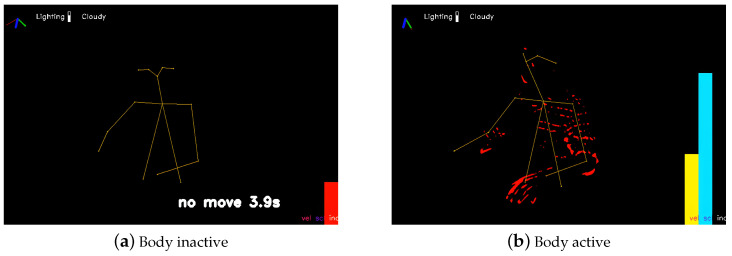
(**a**) Identification of inactive periods where no movement is detected on the subject’s upper body, with the duration of inactivity recorded. The example shows a detected inactive period lasting 3.9 s (red bar). (**b**) Detection results for body movement scale and average 2D movement speed. Visualization of the detected large varying pixels (red dots), movement scale (yellow bar), and average 2D movement speed (blue bar). Motion features are calculated at the pixel level using optical flow to effectively capture subtle movements (e.g., finger motion) during monitoring. The body pose estimation is only for demonstration purposes.

**Movement Speed:** The average 2D speed of body movement per frame is measured as the mean rate of change in pixel positions.

(3) $$\mathrm{Movement}\;\mathrm{Speed}=\frac{1}{n}\sum_{i=1}^{n}S_{i},$$

where $S_{i}$ is the 2D movement speed of pixel *i*, calculated as the magnitude at each pixel of the dense optical flow [29], and *n* is the total number of pixels with significant variations across all RGB channels on the human body [28], as illustrated in red in Figure 3b.

Examples of detections of the features are shown in Figure 3. The three feature statistics were computed on a per-minute basis, then averaged hourly. Minutes in which the participant was present for less than 40% of the time and hours in which the participant was present for less than 40% of the minutes were discarded.

**Statistical Test:** *T* tests were conducted to compare fasting and non-fasting statistics for each participant. The null hypothesis states that there is no significant behavioral change between fasting and non-fasting periods. Welch’s *t* test, which does not assume equal variances between the two groups, was used to analyze the differences between the two conditions due to the imbalanced sample sizes of the fasting and non-fasting groups.

The samples for Welch’s *t* test included all hourly features from fasting and non-fasting days, with sample sizes ranging from 9 to 24 in each group. The *t* test can be represented as

(4) $$T(H_{\mathrm{normal}},H_{\mathrm{fast}}),$$

where $H_{\mathrm{normal}}$ is the set of hourly feature data for non-fasting days and $H_{\mathrm{fast}}$ is the set of hourly feature data for fasting days. A discussion of the statistical significance of the performance achieved by individuals based on the *p* values of the *t* tests presented in Table 2 is included in the next section.

**Energy Expenditure:** When comparing energy expenditure during fasting and non-fasting periods within the same individual, the following assumptions are made: Faster movements generally require more energy, so speed positively contributes to energy expenditure. Larger movements (i.e., greater movement scale) typically involve more muscle groups and, therefore, require more energy, also contributing positively to energy expenditure. Inactivity (rest) results in less energy being expended, contributing less to overall energy expenditure.

Although energy expenditure is influenced by individualized factors such as age, gender, height, and weight [32], for simplicity, a unified equation is applied to all participants to calculate the average hourly energy expenditure here, as follows:

(5) $${\bar{E}}_{\mathrm{loss}\;\left(\mathrm{hour}\right)}=\frac{1}{m}\sum_{t=1}^{m}\left(\alpha \cdot {\mathrm{speed}}_{\mathrm{normalized}}+\beta \cdot {\mathrm{scale}}_{\mathrm{normalized}}+\gamma \cdot {\mathrm{inactivity}}_{\mathrm{normalized}}\right),$$

where α, β, and γ are coefficients that represent the relative contribution of each feature (extracted for each minute) to energy expenditure and *m* is the number of minutes the person is present in the camera’s view. Equations (1)–(3) describe how the raw measurements are computed. All features from each participant are standardized, then normalized to the range of [0, 1] to minimize personal effects when comparing energy expenditure between fasting and non-fasting behaviors.

**Figure 4:**
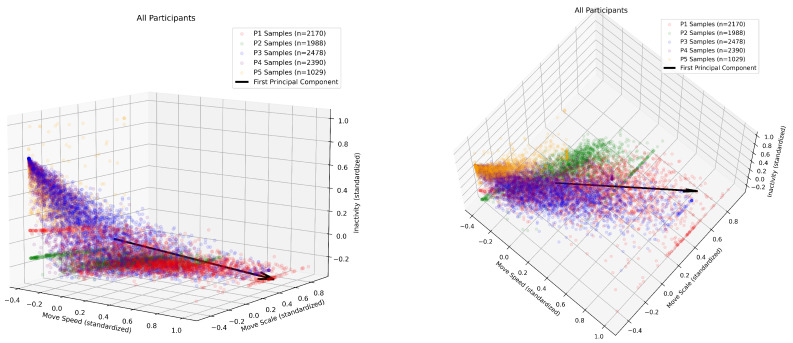
A 3D scatter plot showing the relationship between three standardized features—movement speed, movement scale, and inactivity—for all participants’ samples (n = 10,055 min). (**Left**) side view; (**right**) top view. The black arrow represents the first principal component, which captures the direction of maximum variance in the data. The features are correlated, with movement speed and movement scale showing a strong positive correlation (0.742), while movement speed and inactivity (−0.612), and movement scale and inactivity (−0.763) exhibit strong negative correlations.

A one-minute window is selected to compute all features. Figure 4 shows the correlation between the features and the first principal component derived from Singular Value Decomposition (SVD) [33]. This suggests that faster and larger movements are associated with lower levels of inactivity. The coefficients from Equation (5) are assigned using the absolute values of the normalized first principal component coefficients, i.e., α=0.41, β=0.39, and γ=0.20.

## 4. Results

**Figure 5:**
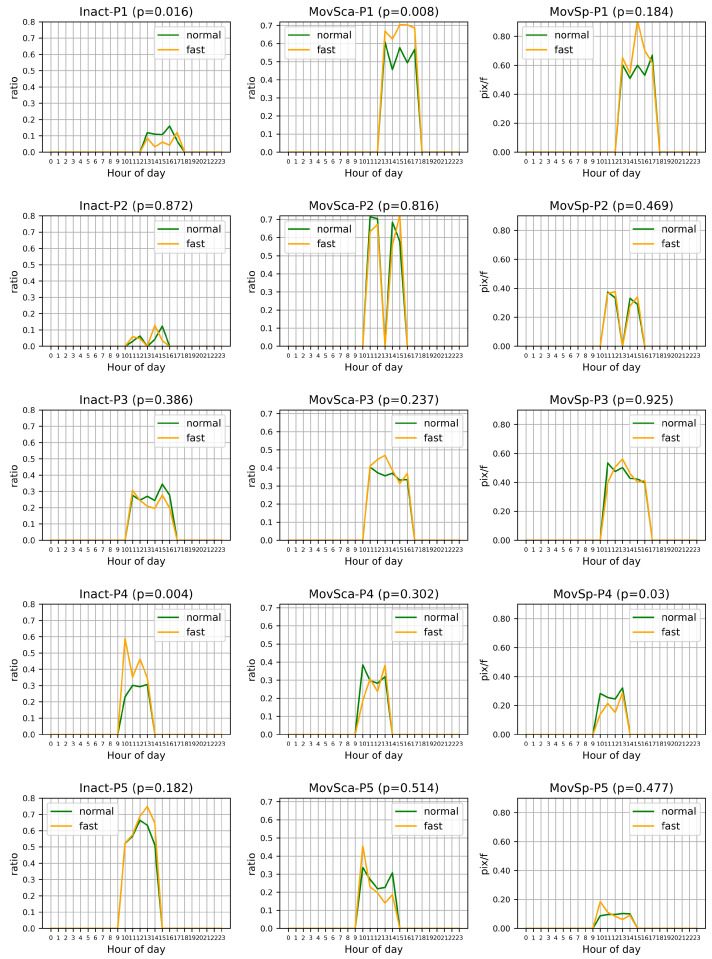
Comparison of hourly averaged inactivity (Inact), movement scale (MovSca), and movement speed (MovSp) for five participants (P1–P5) during fasting and non-fasting days. The *p* values indicate the significance of differences between fasting and non-fasting conditions. Note that only hours with at least four appearances by participants across all days were included in the comparison to avoid bias.

Figure 5 illustrates the movement statistics across different hours of the day for each participant. The analysis focused on three motion descriptors: (i) inactivity, (ii) movement scale, and (iii) movement speed. These descriptors were averaged for both fasting and non-fasting days. To ensure reliability and avoid bias, any hour with fewer than two samples from either the fasting or non-fasting group was discarded. Only hours with at least four appearances by participants across all days were included in the comparison.

This analysis is particularly relevant in Ramadan fasting, where the fasting period is synchronized with daylight hours, typically from sunrise to sunset, which aligns with participants’ daily activity patterns. The monitored data primarily cover the hours of 9:00 to 18:00, reflecting the period when participants were in the lab during normal working hours (although working hours are typically reduced in most Muslim countries during Ramadan [34]). During these hours, the fasting period was still ongoing, making this time window particularly relevant for comparing movement patterns between fasting and non-fasting conditions.

Participant 1 (P1) exhibited increased activity levels during fasting days compared to non-fasting days. This participant showed significantly less inactivity across almost all hours during fasting (p<0.05), a markedly larger upper body movement scale throughout the day (p<0.05), and a faster movement speed during most hours of fasting.

Participant 3 (P3) displayed a similar pattern to P1, being more active on fasting days; however, this was not statistically significant. General declines in movement speed and scale over the day were evident, possibly due to energy depletion without compensatory intake during fasting.

Participant 4 (P4), in contrast to P1 and P3, was notably less active during fasting days, exhibiting significantly more inactivity (p<0.05), an obviously reduced movement scale in the morning, and slower movement throughout the day (p<0.05).

Participant 2 (P2), who engaged in intermittent fasting for non-consecutive days, showed no significant differences between fasting and non-fasting days. For Participant 5 (P5), there was a slight increase in inactivity, suggesting more rest during fasting days, though it was not statistically significant. The scale of body movements followed similar trends during both fasting and non-fasting periods, showing a decrease throughout working hours. However, these differences were also not statistically significant.

In conclusion, P1 demonstrated significant changes in both inactivity and movement scale during fasting, while P4 showed significant changes in inactivity and movement speed. P2, P3, and P5 did not exhibit significant changes across these metrics. For the significantly different features, the changes are consistent across hours of the day, indicating that fasting influences these behavioral aspects.

**Figure 6:**
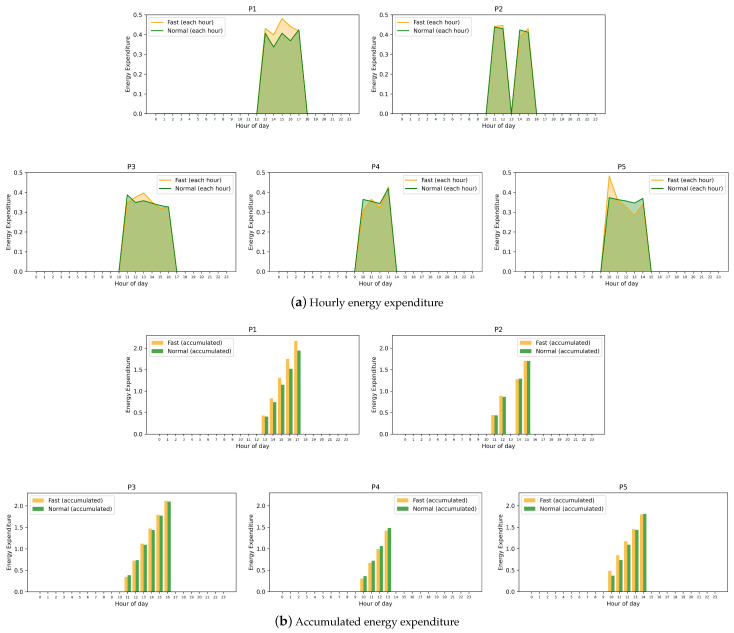
(**a**) Hourly energy expenditure for participants P1–P5 estimated at different hours (averaged across days). (**b**) Accumulated hourly energy expenditure for participants P1–P5 over time (averaged across days).

Figure 6 shows the energy expenditure of each participant derived using Equation (5). For participants without significant behavioral changes (P2, P3, and P5), although there are differences between fasting and non-fasting energy expenditure at certain hours (Figure 6a), the accumulated energy expenditure averaged across all days (Figure 6b) remains very close. P1 shows a noticeable increase in accumulated energy expenditure during fasting, indicating more physical activity during this period. In contrast, P4 shows reduced energy expenditure during fasting hours, with a noticeable dip compared to non-fasting periods, suggesting reduced physical activity.

Overall, the results are mixed across participants and movement features. Although two participants showed significant changes in movement, their responses were opposite.

**Figure 7:**
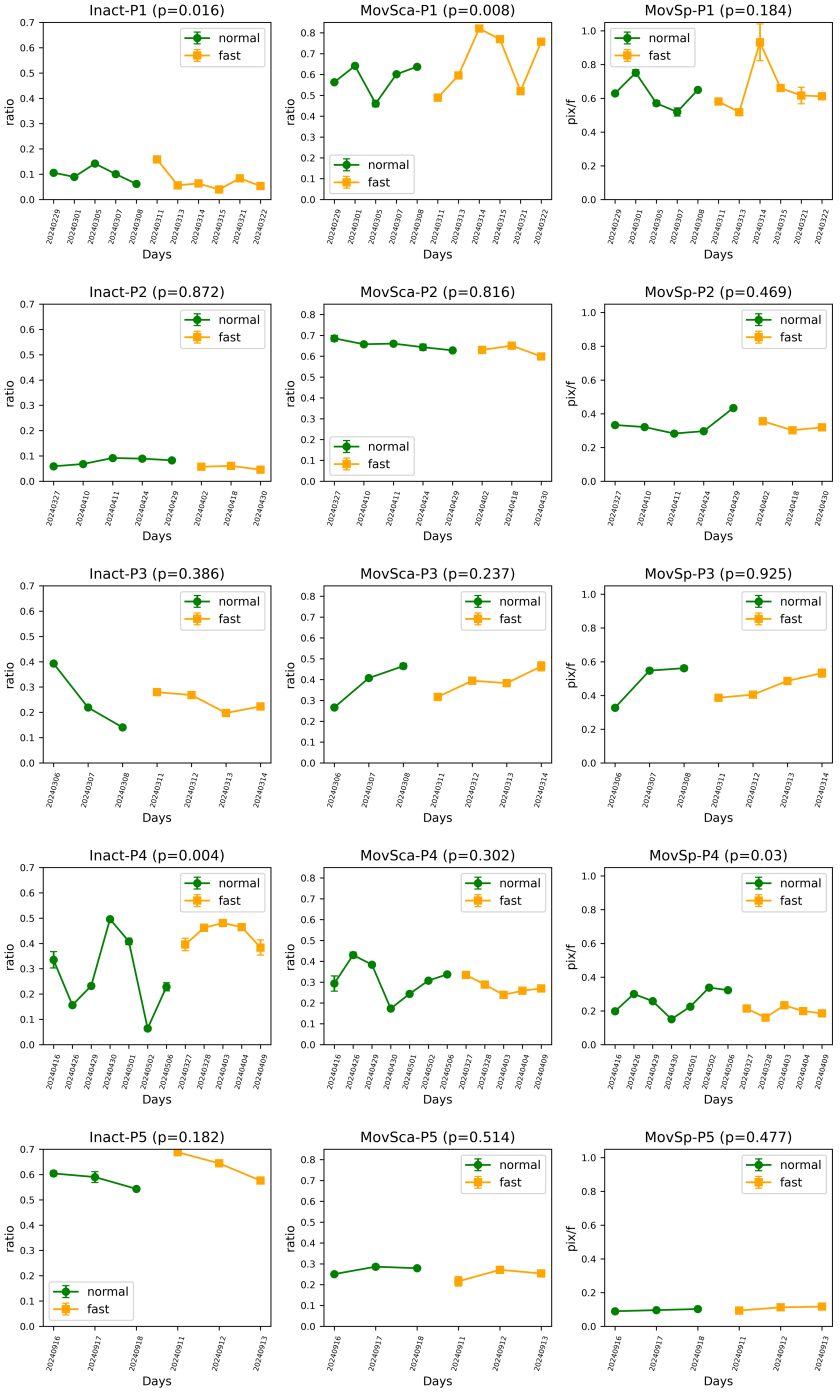
Daily comparison of inactivity (Inact), movement scale (MovSca), and movement speed (MovSp) for five participants (P1–P5) during fasting and non-fasting days (dates when the participant appeared for less than 3 h were discarded). Each plot represents the daily means of these features. The *p* values indicate the significance of differences between fasting and non-fasting conditions.

**Figure 8:**
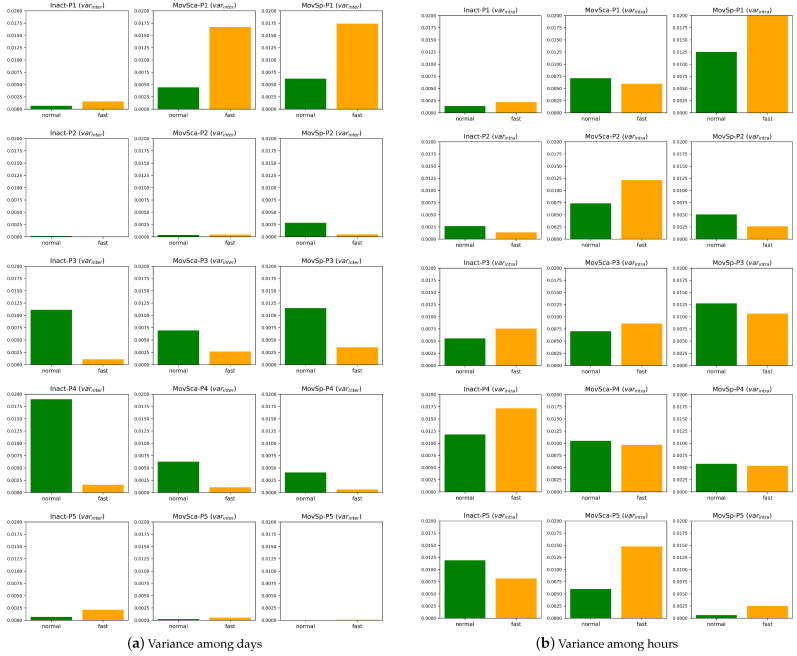
Inter-day (**a**) and intra-day (**b**) variance in features (inactivity (Inact), movement scale (MovSca), and movement speed (MovSp)) across fasting and non-fasting days for participants P1–P5, showing the variation among days and variation within days (among hours).

Figure 7 compares movement feature across each day, averaged across all hours. A clear difference is observed in the statistically significant groups based on daily means. Figure 8 illustrates (a) the variance among days (inter-day variance) and (b) the variance among hours (intra-day variance). Non-fasting days exhibit a typically larger variance compared to fasting days among most of the participants (Figure 8a), indicating that movement behaviors are more consistent during fasting. This consistency could be due to reduced energy levels leading to more uniform behavior throughout the fasting period. However, the variance among hours (Figure 8b) does not show any clear trend when comparing normal and fasting days, which could be because intra-day fluctuations are driven more by task demands or environmental factors than fasting status. It shows that the variance among hours is generally greater than that among days, suggesting that monitoring over longer periods can provide more consistent and reliable statistics for the participants.

## 5. Discussion

The visual monitoring system proposed in this research offers significant benefits by examining the effects of fasting on upper body movement and energy expenditure. It enables non-invasive, continuous, and objective data collection, allowing for natural observation without disrupting participants’ routines. The system, equipped with an RGB-D camera and a compact processor, provides high-resolution spatial and temporal data processed in real time, capable of capturing subtle body movements. The RGB-D camera, with its infrared-based depth measurement, is well suited for robust human detection in various home environments, especially in low-light conditions, compared to standard RGB cameras [28]. Its automation and scalability make it suitable for larger studies, while its cost-effectiveness enhances applicability in diverse settings. By tracking movement over multiple fasting and non-fasting days, the system offers insights into behavioral adaptations during fasting in professional environments. Although fasting was the control variable here, the approach might also be usable for assessing the impact of different approaches to dieting.

The impact of fasting on upper body movement during office work did not show a consistent trend among the five participants, highlighting significant interpersonal differences. According to Table 1, the five participants, despite variations in age and BMI, have similar lifestyles. Among all participants, P2 showed the least changes in all motion features (less than 6%), primarily due to intermittent fasting (similar to P5, who exhibited no significant change despite being in a different age group, though P5’s changes were still twice as large as P2’s). Age may be a factor here, but this study considered too few participants to draw definitive conclusions.

For P3 and P5, who are close in age to P4, the observed differences in the experimental results may stem from their varied fasting experiences and habits. P5, for example, engaged in three daytime fasts outside of Ramadan, a relatively short period that may not have been sufficient for significant fasting effects to emerge. In contrast, P3 not only fasted long-term during Ramadan but also maintained a regular practice of fasting one or two days weekly throughout the year. This consistency likely contributed to higher adaptability to fasting in P3’s body. Meanwhile, P4 fasted only during Ramadan, suggesting less frequent exposure to fasting conditions. It is also possible that upper body monitoring is not as sensitive to fasting, especially compared to walking or exercise monitoring. However, in such cases, individuals are unlikely to engage in these activities for the entire daytime.

The opposite changes between P1 and P4 could be attributed to (i) personal differences and habits, with the inconsistency matching previous studies that indicate fasting has different effects on different people, and (ii) the content of work, where differing tasks and patterns seem to be related factors; for example, P1 mentioned working primarily on programming with a PC and felt that their working hours were somewhat reduced, though they did not notice any significant changes in their daily routine. In contrast, P4, who focused mostly on online meetings, hands-on experiments, and data analysis, reported difficulty concentrating during fasting due to lack of sleep, frequent headaches, dry mouth, and a need for more naps and rest.

People may adjust their working hours during Ramadan, often shifting to shorter hours or different timings to accommodate fasting and prayer times (e.g., staying up late to ensure a meal before sunrise), which can impact their energy, focus, and productivity levels [35]. Regarding changes in working hours, the mean working hours during fasting and non-fasting periods are shown in the first row of Table 3. The average number of hours spent at a desk is 5.07 (SD 1.45) during non-fasting and 5.18 (SD 1.2) during fasting. This indicates that the average change in working hours is less than an hour for all participants. For the primary monitoring period, from 9:00 to 18:00 for all participants, the start and end working times were compared, and no shifts from their normal working routines during fasting were observed, suggesting that adjusted working hours were not a significant factor in this research.

Other underlying personal variations could also play a role, including differences in genetics, metabolic rate, hormone levels, stress levels, and previous diet. These factors can affect how individuals respond to fasting, which may explain the differences seen among participants.

The limitations of this research include (i) the small size of the dataset, which limits diversity in terms of gender and age and affects the generalizability of fasting’s impact on individuals; (ii) the fact that observation was restricted to desk work and upper body movements during working hours, failing to capture full-day energy expenditure; and (iii) the fact that the experimental setup was a natural observation, where participants were monitored on different dates, and their monitoring periods were not necessarily continuous due to participants’ absences from the workplace. Conducting longer-term research, beyond just a few days, would provide deeper insights into how behavior changes over extended fasting periods.

## 6. Conclusions

This research aimed to examine the impact of fasting on upper body movement patterns in daily office and sitting environments. Results from the five participants highlight the variability in fasting’s effect on physical activity. Significant changes observed in some participants suggest that fasting can influence movement behavior, while the lack of substantial changes in others indicates that the effects of fasting may be highly individualized. These findings underline the importance of considering personal variability when studying the effects of fasting on movement and energy expenditure.

Future studies should include a larger sample size and more diverse participant profiles. Additionally, future work should incorporate more fine-grained measurements of movements in different body parts to analyze the effects of fasting on various subsystems, such as the head or hands. Post-fasting recovery monitoring, focusing on how physical activity and movement recover or change after fasting periods, could also help in understanding the long-term impact of fasting on health and energy levels.

## Figures and Tables

**Table 1:** Participant profiles showing an overview of the participants’ demographic information and lifestyle factors. Abbreviations: P—participant; GE—gender; AG—age; MC—medical conditions; DA—drinking alcohol; SM—smoking; BMI—Body Mass Index; FD—fasting during Ramadan (RMD)/non-Ramadan (N-RMD).

P	GE	AG	MC	DA	SM	BMI	FD
P1	Male	29	No	No	No	21.3	RMD
P2	Male	70	No	Rarely	No	26.2	N-RMD
P3	Male	40	No	No	No	32.6	RMD
P4	Male	37	No	No	No	26.8	RMD
P5	Male	35	No	No	No	20.7	N-RMD

**Table 2:** *p* values from Welch’s *t* tests comparing hourly features (inactivity, movement scale, and movement speed) between non-fasting and fasting days for each participant (P1–P5). Lower *p* values (p<0.05, bold) indicate a statistically significant difference in behavior between the two periods.

Participant	Inactivity	Movement Scale	Movement Speed
P1	**0.016**	**0.008**	0.184
P2	0.872	0.816	0.469
P3	0.386	0.237	0.925
P4	**0.004**	0.302	**0.030**
P5	0.182	0.514	0.477

**Table 3:** Detailed changes between fasting and non-fasting periods for five participants (P1–P5). The table presents working hours and percentage changes—mean hourly value differences during the fasting period (Fast) compared to the non-fasting period (Normal)—in inactivity (Inact), movement scale (MovSca), and movement speed (MovSp).

	P1		P2		P3		P4		P5	
	Normal	Fast	Normal	Fast	Normal	Fast	Normal	Fast	Normal	Fast
**Hours**	6.0	5.0	4.5	5.0	6.67	7.25	3.2	4.0	5.0	4.67
**Inact**	100%	−40.1%	100%	−5.1%	100%	−12.8%	100%	+45.9%	100%	+9.7%
**MoveSca**	100%	+24.6%	100%	−1.4%	100%	+10.3%	100%	−12.5%	100%	−9.8%
**MoveSp**	100%	+17.0%	100%	+5.5%	100%	−0.9%	100%	−25.4%	100%	+11.7%

## Data Availability

The data presented in this study are available on request from the corresponding author due to privacy and ethical reasons.
